# Tubular bile duct structure mimicking bile duct morphogenesis for prospective in vitro liver metabolite recovery

**DOI:** 10.1186/s13036-020-0230-z

**Published:** 2020-03-19

**Authors:** Astia Rizki-Safitri, Marie Shinohara, Minoru Tanaka, Yasuyuki Sakai

**Affiliations:** 1 Department of Bioengineering, Graduate School of Engineering, The University of Tokyo, Tokyo, Japan; 2 Center for International Research on Integrative Biomedical Systems (CIBiS), Institute of Industrial Science (IIS), The University of Tokyo, Tokyo, Japan; 3 Department of Chemical System Engineering, Graduate School of Engineering, The University of Tokyo, Tokyo, Japan; 4 Laboratory of Stem Cell Regulation, Institute for Quantitative Biosciences (IQB), The University of Tokyo, Tokyo, Japan; 5 Department of Regenerative Medicine, Research Institute, National Center for Global Health and Medicine (NCGM), Tokyo, Japan; 6 Max Planck-The University of Tokyo, Center for Integrative Inflammology, The University of Tokyo, Tokyo, Japan

**Keywords:** Bile duct, Morphogenesis, Liver metabolite, Bile recovery, Hierarchical co-culture

## Abstract

**Background:**

Liver metabolites are used to diagnose disease and examine drugs in clinical pharmacokinetics. Therefore, development of an in vitro assay system that reproduces liver metabolite recovery would provide important benefits to pharmaceutical research. However, liver models have proven challenging to develop because of the lack of an appropriate bile duct structure for the accumulation and transport of metabolites from the liver parenchyma. Currently available bile duct models, such as the bile duct cyst-embedded extracellular matrix (ECM), lack any morphological resemblance to the tubular morphology of the living bile duct. Moreover, these systems cannot overcome metabolite recovery issues because they are established in isolated culture systems. Here, we successfully established a non-continuous tubular bile duct structure model in an open-culture system, which closely resembled an in vivo structure. This system was utilized to effectively collect liver metabolites separately from liver parenchymal cells.

**Results:**

Triple-cell co-culture of primary rat hepatoblasts, rat biliary epithelial cells, and mouse embryonic fibroblasts was grown to mimic the morphogenesis of the bile duct during liver development. Overlaying the cells with ECM containing a Matrigel and collagen type I gel mixture promoted the development of a tubular bile duct structure. In this culture system, the expression of specific markers and signaling molecules related to biliary epithelial cell differentiation was highly upregulated during the ductal formation process. This bile duct structure also enabled the separate accumulation of metabolite analogs from liver parenchymal cells.

**Conclusions:**

A morphogenesis-based culture system effectively establishes an advanced bile duct structure and improves the plasticity of liver models feasible for autologous in vitro metabolite-bile collection, which may enhance the performance of high-throughput liver models in cell-based assays.

## Background

The bile duct demonstrates metabolite accumulation and is involved in extracellular transport from the liver parenchyma [1]. Metabolites in bile contain vital information related to chemical and drug metabolism [2–4] including the histories of liver-related disease such as liver cholestasis [4, 5]. The presence of the bile duct in a liver culture model would enable bile recovery and enhance the relevancy of cell-based hepatotoxicity assays. However, this liver model has not been considered, as no bile duct models can recover liver metabolites from the culture system.

As a major approach for establishing a bile duct model, liver differentiation experiments may generate in vitro bile ducts from induced pluripotent stem cells following downstream hepatoblast differentiation [6–9]. This bile duct is mainly evaluated as cyst formation and is established in an embedded-extracellular matrix (ECM) such as Matrigel [6, 7, 9]. Its differentiation is enhanced by fibroblasts [6, 7] and cytokines such as hepatocyte growth factor and transforming growth factor beta 1 (TGF–β1) [10, 11]. Although the formed cyst functionally corresponds to bile ducts, there are morphological differences between the cyst and bile duct structure. The authentic bile duct consists of highly polarized cholangiocytes/biliary cells (BEC) in a tubular structure [12]. Moreover, the ECM-embedded culture does not interact with liver parenchymal cells/hepatocytes, which are the primary bile-producing cells.

Alternatively, an intrahepatic biliary duct (IHBD) which is directly connected to the hepatocyte, can be developed in a culture system [13]. The IHBD shares an identical progenitor lineage with hepatocytes and oval cells [14, 15]. During rat liver development, IHBD is significantly assembled from the bile duct cysts surrounding the hepatic portal vein between E.15 and neonate age [16–18]. The mechanism underlying tubulogenesis in IHBD is complex and depends on signaling from neighboring tissues. This process includes several steps such as the appearance of ductal plates [15, 16], hepatoblast fate determination by *Notch2* activation [15, 19, 20], *Jagged1* expression by mesenchymal cells in the portal vein [15, 16, 21], and cytokine–triggered differentiation [16]. This mechanism distinguishes bile duct tubulogenesis from the formation of other tubular tissue such as blood vessels and kidney tubules [22]. Though this process has been extensively analyzed in animal models, there is no report, to our knowledge, confirming its reproducibility in vitro.

Based on these considerations, development of a relevant bile duct model has presented challenges because its complex tubular shape and closed culture limit the application of the bile duct model, and functioning depend on liver parenchymal cells. Here, we propose a tubular bile duct structure using a triple liver cell co-culture for simulating IHBD morphogenesis, employing rat hepatoblasts, rat BECs, and mouse embryonic fibroblasts (MEFs). This structure offers superior morphology to currently available bile duct cysts. This model also provides an open-culture system that permits hepatobiliary interaction and metabolite accumulation in the bile duct structure by using a collagen culture insert. We demonstrated that the advanced bile duct culture favorably enhanced the performance of liver models for various purposes, particularly in vitro bile recovery.

## Results

### Triple co-culture in high Matrigel ECM content allows establishment of tubular bile duct structures

**Fig. 1:**
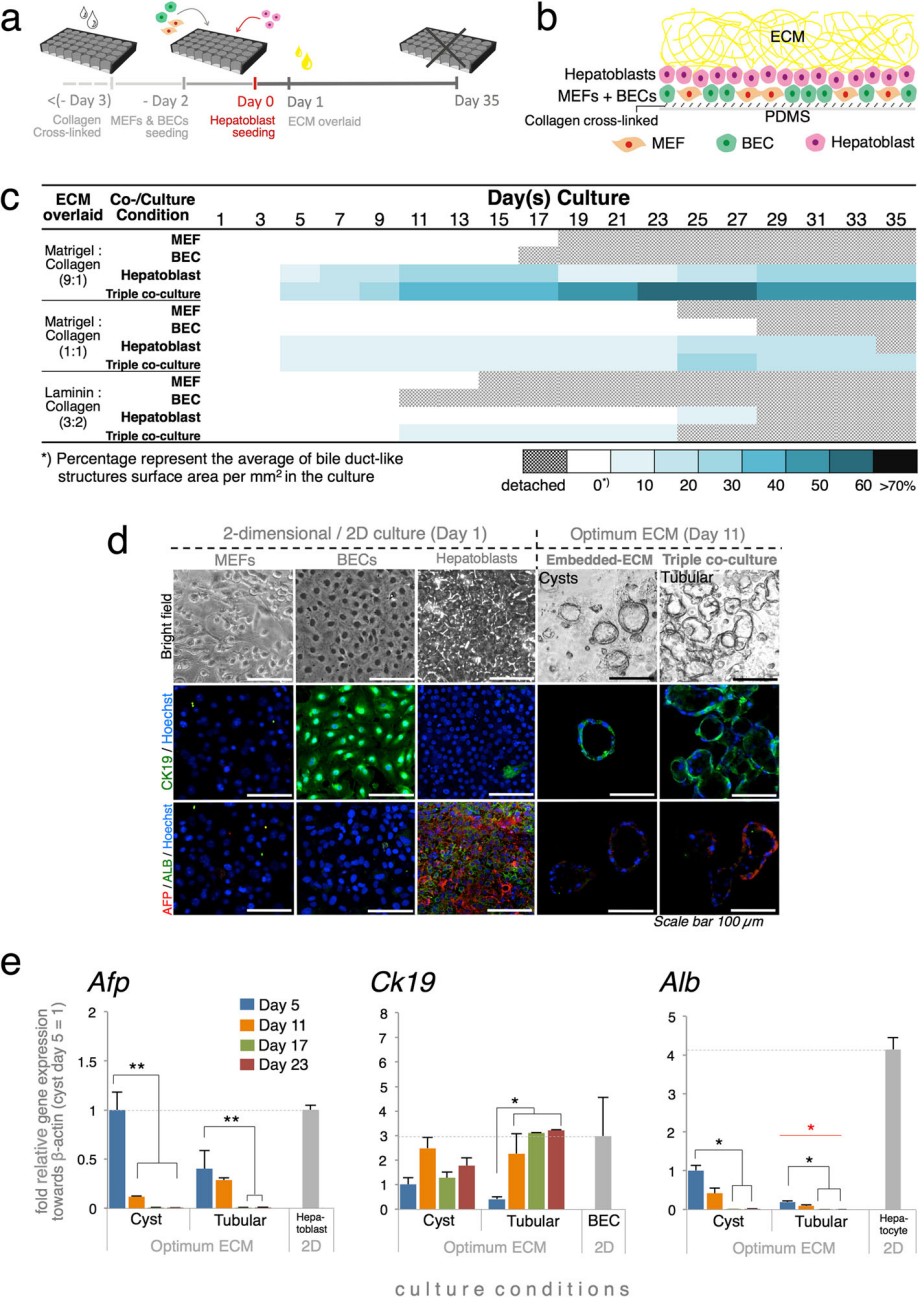
Optimum condition for the establishment of tubular bile duct structure. **a** PDMS–plate treatment and seeding procedure for initial hierarchal co-culture. **b** Diagram showing cross-section of bile duct hierarchal co-culture after ECM overlay. **c** Map showing average % of bile duct-like structure surface area (mm^2^) in all cultures after ≤ 35 days (*n* = 12; three independent experiments). **d** Bile duct cysts and tubular bile duct structures. **e** Gene expression assay comparing bile duct cysts and tubular bile duct structure. Tubular bile duct structures highly expressed *Ck19* marker but only weakly expressed hepatoblast (*Afp*) and hepatocyte (*Alb*) markers. *β-actin* was used as a housekeeping gene and primary cells served as a negative control (*n* = 5; three independent experiments, values are the means ± SD. *P < 0.05; **P < 0.01. Red stars and bars indicate significant differences between bile duct structures)

Previous studies reported that oxygenated culture conditions supported by a poly (dimethylsiloxane) (PDMS)-bottom plate can improve the maintenance of primary hepatocytes in vitro [23, 24]. However, it was unclear whether such a condition is suitable for maintaining the bile duct. Preliminary experiments showed that oxygenation is vital for the establishment of a bile duct structure. Bile ducts cultured on tissue culture polystyrene (TCPS) surfaces developed poorly over a specific time period (Additional file 1; Fig. S1a). We designed and constructed the prototype of a culture layout (Fig. 1a) that promoted organization mimicking the early stages of IHBD tubulogenesis. Three distinct cells were used for co-culture: a hepatoblast (which is a liver progenitor cell), a biliary epithelial cell/BEC, and a mouse embryonic fibroblast/MEF (which is a type of mesenchymal cell widely used to induce BEC differentiation in BEC-derived induced pluripotent stem cells) [6, 7]. We used mitomycin-treated MEFs that have a low proliferation rate. The ECM was overlaid 1 day after hepatoblast seeding and a finely aligned hierarchal culture was produced (Fig. 1b). Subsequent modulations of the cell proportion and ECM composition were performed to determine the optimum conditions for the culture system to establish tubular structures.

To evaluate the bile duct structure, the bile duct luminal area was calculated (Additional file 1; Fig. S1b-c). A triple co-culture, MEF-BEC-hepatoblast system was preferable for bile duct structure establishment. The bile duct structure was rapidly established in a culture containing an ECM mixture consisting of Matrigel and collagen 9:1 (Fig. 1c; Additional file 1; Fig. S1c). The bile duct structure was also observed in an ECM mixture consisting of Matrigel and collagen 1:1 but was dominated by sinusoidal-like structures (Additional file 1; Fig. S1d). The bile duct-like structures observed in these ECM mixtures were sustained for ≤ 35 days. An ECM mixture composed of 3:2 laminin and collagen gel [9] was also tested because laminin has been reported to significantly alter bile duct differentiation [6, 9]. However, far fewer bile duct structures were observed in this laminin–collagen mixture than in the other matrices as it could no longer support durable tissue attachment after 10 days. In our models, bile duct structures were observed only in monocultures and co-cultures containing hepatoblasts but were absent in the BEC monoculture and BEC-MEF co-culture regardless of ECM composition (Additional file 1; Fig. S1b).

To evaluate the optimum ECM composition, bile duct-like structure development was compared between the triple co-culture and standard embedded-Matrigel culture. Two types of bile duct structures were observed: cysts with lumen forming round sac-like structures and tubular structures with lumen organized in a non-continuous network-like formation (Fig. 1d). The former became established in the embedded-Matrigel culture and was frequently observed in the hepatoblast culture-overlaid optimum ECM. The tubular structure frequently developed in the triple co-culture–overlaid optimum ECM.

Immunostaining and gene expression analyses revealed that the tubular structure established in triple co-culture expressed a stronger indication of hepatoblast differentiation into the bile duct (Fig. 1d, e). The tubular structures expressed lower levels of the hepatoblast marker alpha-fetoprotein (*Afp*) [25] than cysts after 11 days of culture. *Afp* in the tubular structure had significantly downregulated prior to 5 days of culture followed by upregulation of the BEC marker keratin 19 (*Ck19*) [6, 7, 9]. A substantially low expression level of the hepatocyte marker albumin (*Alb*) [23, 25] in the cysts and tubular structures relative to that in hepatocytes confirmed hepatoblast differentiation into biliary cells.

### Tubular bile duct structure established in triple co-culture exhibits improved maturity, polarity, and accumulation capability

**Fig. 2:**
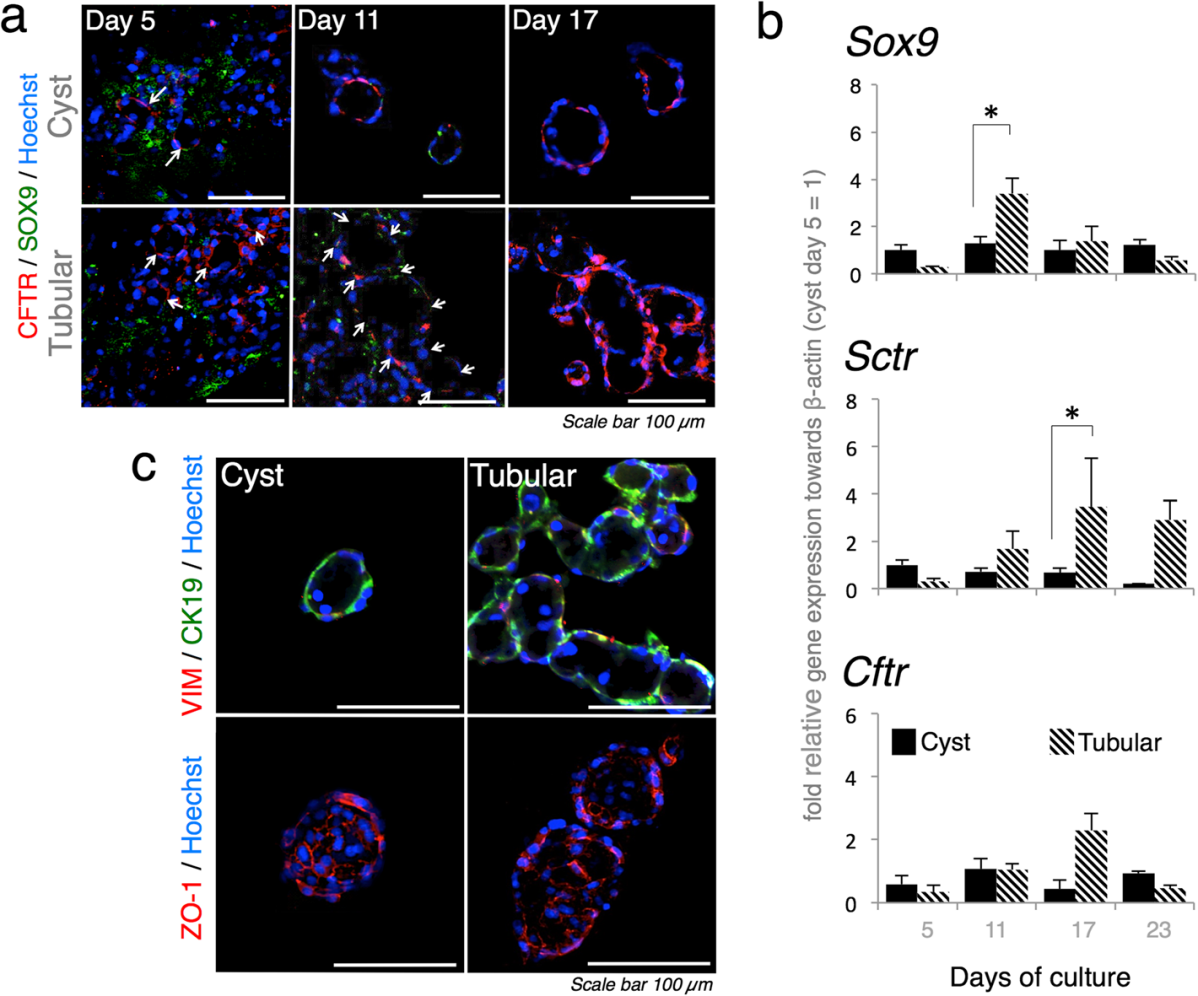
Characteristics of tubular bile duct structure established in triple co-culture in ≤ 23 days. **a** Tubular bile duct structure in triple co-culture expressing significant levels of the bile duct markers *Sox9*, *Cftr,* and *Sctr*. (*β-actin* was used as the housekeeping gene, *n* = 5; three independent experiments; values are the means ± SD; *P < 0.05). **b** Differentiation stages in cyst and tubular bile duct structure. White arrows emphasize the shape. Stages were validated by immunostaining with SOX9 and CFTR at ≤ 17 days. **c** Immunofluorescence shows the 17 day tubular bile duct slightly expressing VIM and resembling fine ZO1 tight junctions

To evaluate the maturity of the tubular bile duct structure, three BEC markers were used: SRY-box 9 (*Sox9*), secretin receptor (*Sctr*) and the cystic fibrosis transmembrane conductance regulator (*Cftr*) [6, 7]. The tubular structure in the triple co-culture was highly differentiated from the cyst, as shown by significant changes in *Sox9* and *Cftr* expression (Fig. 2a). High expression levels of *Sctr* were inversely correlated with downregulated *Sox9* expression in the tubular structure after 11 days of culture. In addition to *Sctr*, the significant induction of *Cftr* expression of the tubular structure suggested that maturation of the bile duct progressed at approximately 11 days post co-culture (Fig. 2b) and up to 23 days. Several tubular structures slightly expressed vimentin (*Vim*), which is a specific mesenchymal cell marker [27, 28]. Both the tubular and cystoid structures resembled a fine tight junction as shown by zonula occludens 1 (*Zo1*) [7] (Fig. 2c).

**Fig. 3:**
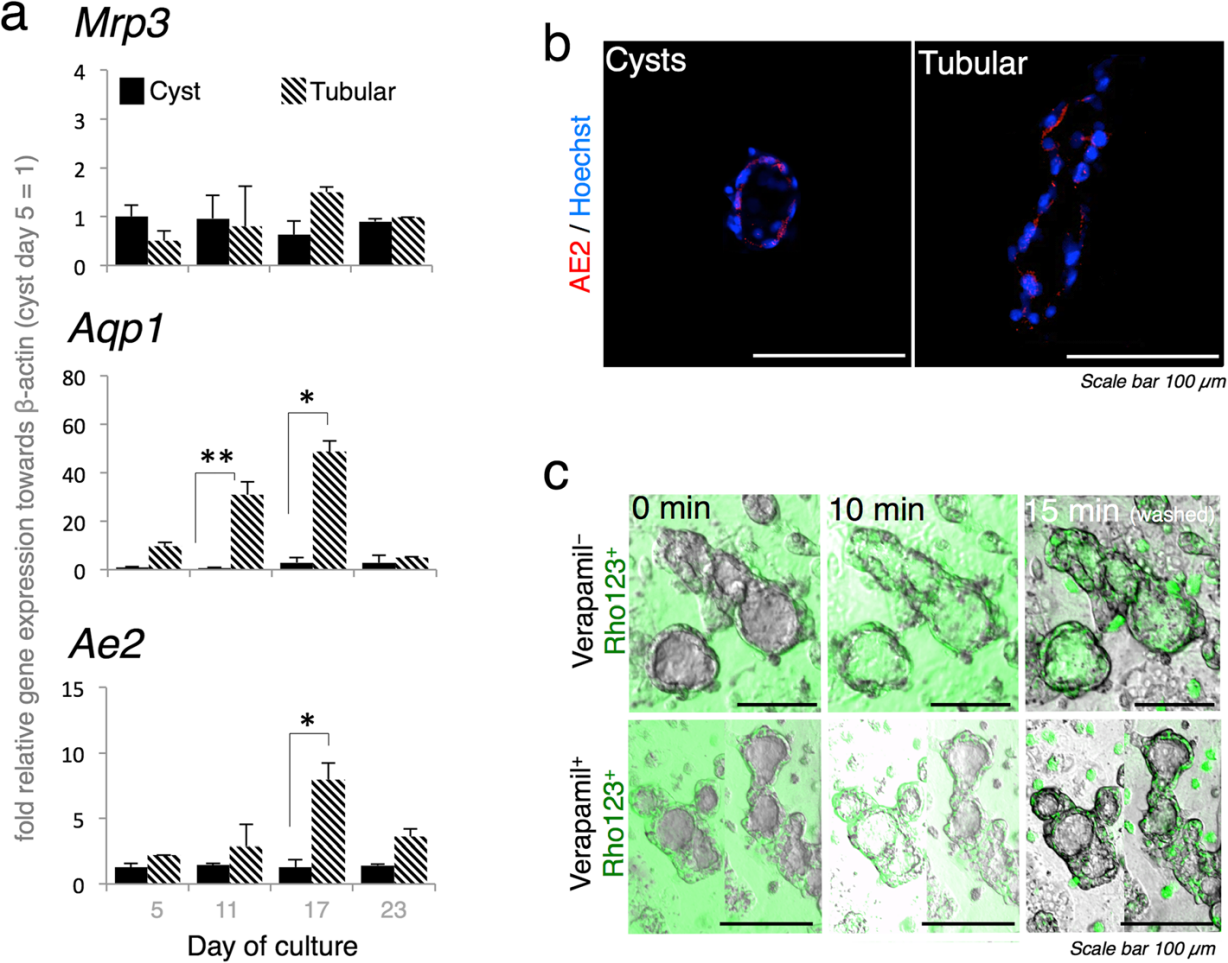
In vitro ≤ 23 day triple co-culture, tubular bile duct structure polarity and transporter activity. **a***Aqp1* and *Ae2* were significantly upregulated in the tubular structure whereas *Mrp3* expression did not significantly change (*β-actin* was used as the housekeeping gene, n = 5; three independent experiments; values are means ± SD. *P < 0.05; **P < 0.01). **b** AE2 was abundantly distributed on the apical site. **c** Rhodamine 123 accumulated in the lumen of the tubular structure. Verapamil blocked MDR1 transporter activity and attenuated green fluorescence in the lumen

The bile duct is composed of epithelial cells with a cell polarity characterized by the expression of localized proteins at the basolateral and apical sites. These sites contain specific transporters associated with bile duct function [22]. Multidrug resistance receptor 3 (*Mrp3*) [29], a basolateral membranous transporter, was constitutively expressed under all culture conditions. Meanwhile, the tubular structure in the triple co-culture showed higher secretion levels than the cysts for aquaporin 1 (*Aqp1*) [7] at day 11 and anion exchange 2 (*Ae2*) [29] activity at day 17 (Fig. 3a). Immunofluorescence staining confirmed that the AE2 transporters were on the apical site (Fig. 3b). Along with *Cftr*, the expression of these transporters seemed to decrease at day 23, indicating that the structure was gradually losing its function.

The tubular bile duct structure also responded to certain drugs. Rhodamine 123 is widely used to evaluate the activity of multidrug resistance protein 1 (MDR1), which is expressed mainly in the bile duct and inhibited by verapamil [7, 9, 30]. Time-lapse images taken 15 min after rhodamine 123 was administered demonstrated that it accumulated in the tubular structure. Verapamil addition blocked rhodamine 123 accumulation and reduced green fluorescence in the lumen of the tubular structure (Fig. 3c; Additional file 2; Fig. S2).

### Tubular bile duct structure development in the triple co-culture mimics in vivo bile duct morphogenesis

**Fig. 4:**
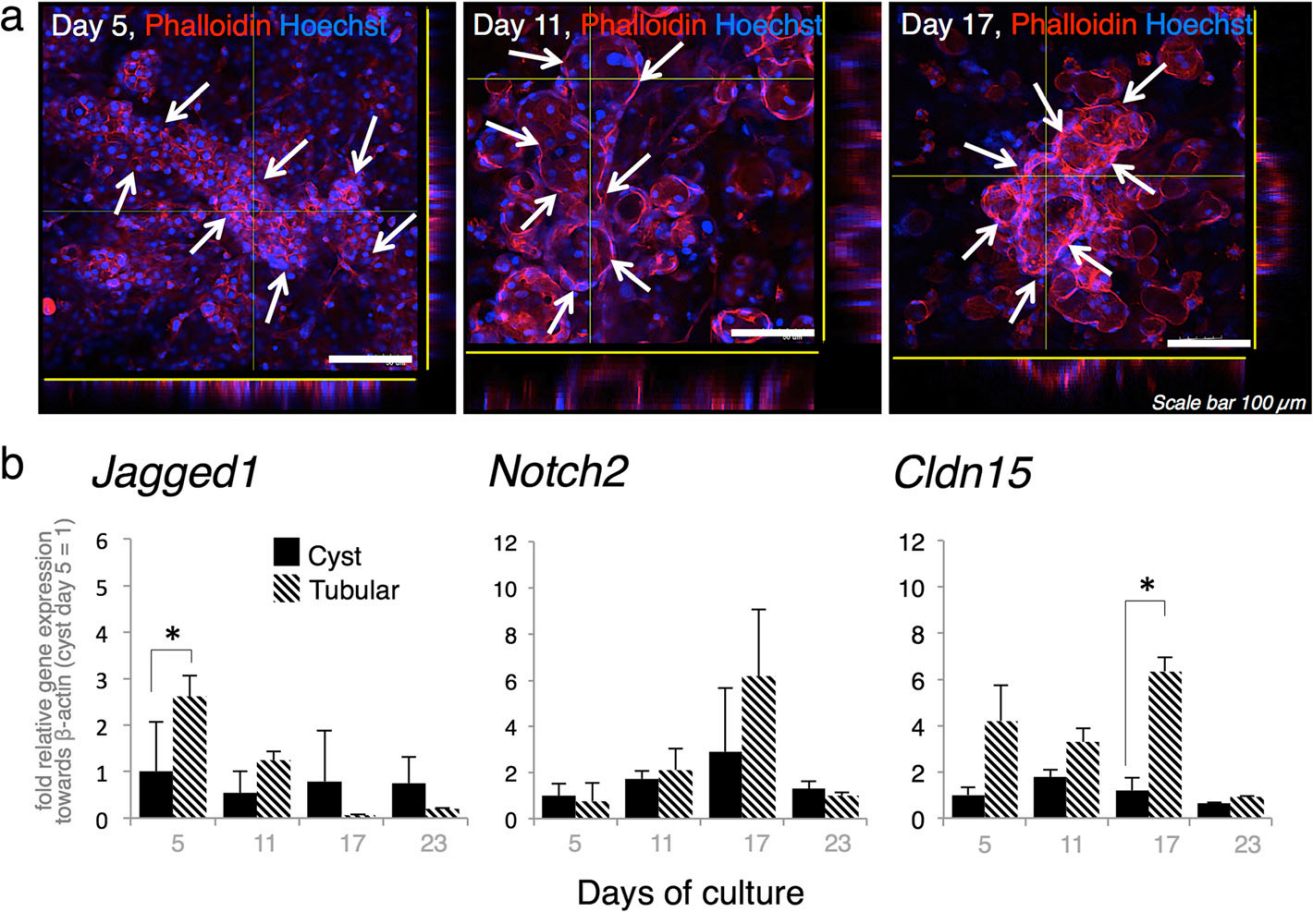
Morphogenesis of tubular bile duct structure established within the 17 day triple co-culture period. **a** Three-sided imagery of phalloidin and Hoechst staining resembling the tubular structure of an in vitro bile duct (white arrow), displaying continuous lumen structure within the tubular structure. **b** Tubular structure showed significant upregulation of *Jagged1*, *Notch2*, and *Cldn15* within 17 days of culture (*β-actin* was used as the housekeeping gene, n = 5; three independent experiments; values are means ± SD; *P < 0.05)


To understand tubular bile duct structure development in the triple co-culture, a real-time recording was made at ≤11 days into the culture. There was rapid morphogenesis of the tubular structure at 7–11 days (Additional file 3: Movie S3). The early stage of morphogenesis in the tubular structures was initiated by multiple tissue mounds at day 5 followed by elongated structures along the neighboring tissue at day 11. The mounds showing hepatoblast morphology were initially surrounded by MEFs and BECs. Actin filament and nuclear staining with phalloidin and Hoechst (Fig. 4a) revealed the transformation of a mound into a tubular structure at ≤ 17 days into the culture. A continuous lumen formed and elongated in the tubular structure according to three-dimensional/3D-volume (Additional file 4: Movie S4) images. We assessed the possible expression of bile duct tubulogenesis signals by measuring *Jagged1* and *Notch2* [15, 20, 21] in the total cDNA extracted from the tissue. The tubular structure presented with significantly higher bile duct tubulogenesis-related marker levels than the cyst structure (Fig. 4b). *Jagged1* was significantly upregulated at day 5 followed by increased in *Notch2* expression starting on day 11.

**Additional file 3: Movie S3.** Three-dimensional images of the tubular bile duct structure at days 5, 11, and 17 s.
**Additional file 4: Movie S4.** Three-dimensional images of the tubular bile duct structure at days 5, 11, and 17 s.


Claudin 15 (*Cldn15*) expression (Fig. 4b) also increased during the activation period and continued throughout culture. This gene is an analog of Claudin 15-like b (*Cldn15lb*) which is a critical tight junction protein for IHBD organization in zebrafish [26]. *Cldn15* was detected in both the cyst and tubular structures but expressed at a relatively higher level in the tubular structures.

### Tubular bile duct structure accumulated liver metabolites from hepatocyte culture

**Fig. 5:**
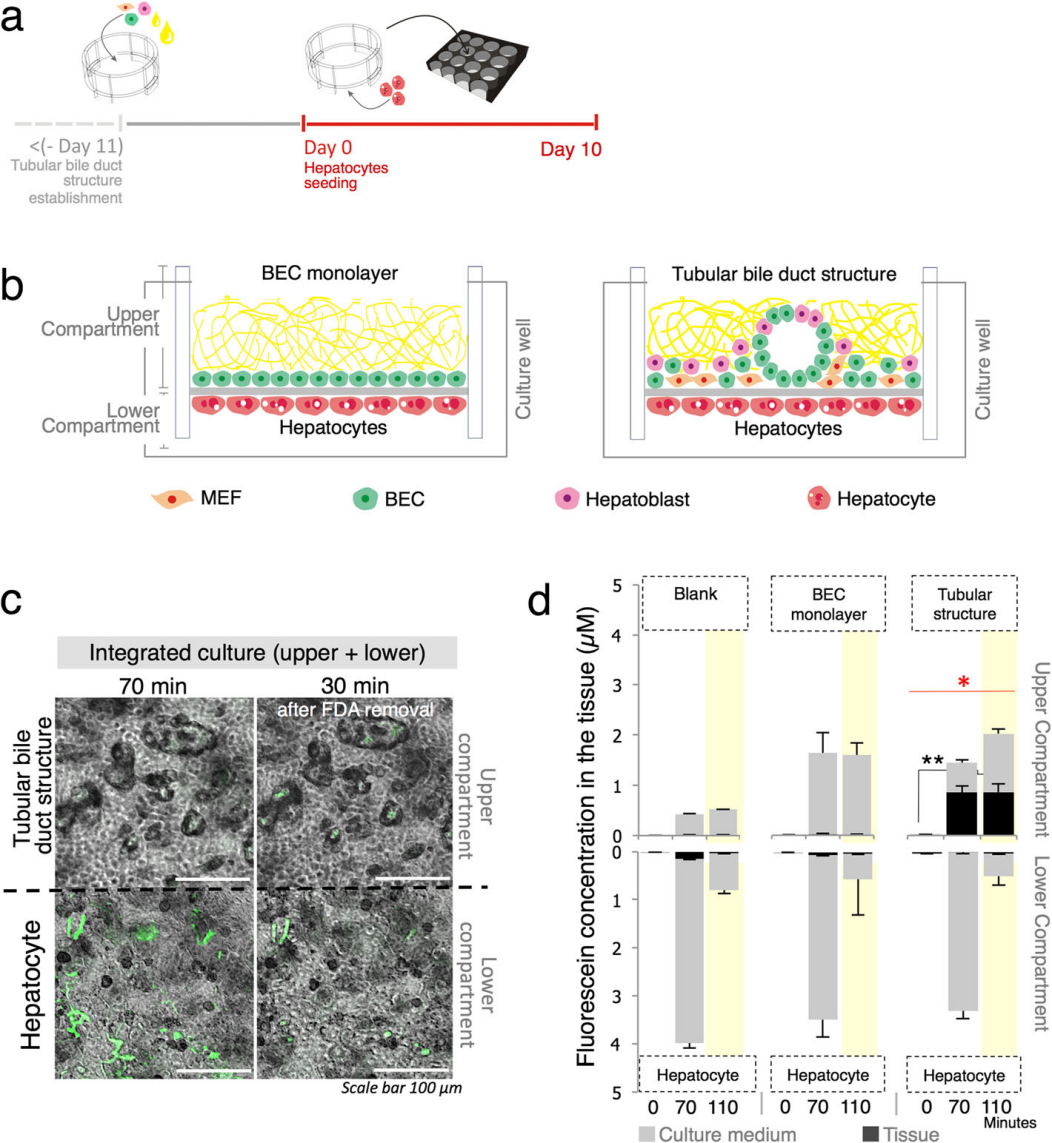
Integrated culture system combining in vitro tubular bile duct structure and primary hepatocyte tissue. **a** Seeding procedure for integrated tubular–hepatocyte culture. **b** Diagram showing hierarchy of integrated BEC-hepatocyte and tubular–hepatocyte culture established in collagen membrane-culture insert. **c** FDA assay administrated to hepatocyte-contained compartment. Fluorescein presented by green fluorescence accumulated in tubular bile duct structures located in the upper compartment. **d** Tubular structures demonstrated the ability to accumulate fluorescein. FDA removal is represented by yellow backlights. (*n* = 4; two independent experiments; values are the means ± SD; *P < 0.05; **P < 0.01). Red stars and bars indicate significant differences between independent treatments)

Given that bile ducts are functionally and structurally critical to hepatocyte physiology [31], we investigated the association between in vitro bile ducts and hepatocyte behavior in culture. A direct co-culture between the tubular bile duct structure and hepatocytes demonstrated possible inhibitions on the viability of both (data not shown). Thus, we used a permeable collagen membrane-culture insert [32] by combining our established tubular bile duct structure with primary hepatocyte culture with BEC monolayer culture used for comparison. This collagen membrane-culture insert not only supported tissue attachment and allowed molecule transportation, but also enhanced the compatibility and scrutability of the combined tissue. Hepatocytes were seeded onto the opposite site of day-11 tubular structure culture, which remained on the lower compartment (Fig. 5a, b; Additional file 5; Figure S5a). This placement prevented detachment and maintained the stability of the tubular bile duct structure. Both tissues were successfully cultured and placed in a 24-well PDMS plate for oxygenation.

We demonstrated in vitro metabolite transport and accumulation from the hepatocytes to the tubular bile duct structure which was quantified by a fluorescein diacetate (FDA) assay [1, 33]. FDA is hydrolyzed by living cells into fluorescein [34] and can be observed by determining the FITC emission spectrum [1]. FDA was added to the lower compartment where the hepatocytes were seeded and the total fluorescence stabilized after 20–30 min incubation (Additional file 5; Fig. S5b). We also observed that all tested tissue generally produced fluorescein, whereas levels were higher in the hepatocyte culture. In the integrated culture system, fluorescein accumulated inside the hepatocyte bile canaliculi after 70 min incubation and was observed in the tubular structure located on the upper compartment at 30 min after FDA removal (Fig. 5c). An assay of fluorescein accumulation showed that the tubular bile duct structures significantly retained fluorescein whereas the BEC monolayer surpassed fluorescein toward the upper compartment. The tubular structures accumulated 0.8 μM fluorescein, which was not substantially cleared after 70 min during FDA removal (Fig. 5d). This phenomenon was neither observed in the BEC monolayer nor in the control cultures containing only hepatocytes. The control culture showed that FDA removal reduced the amount of fluorescein retained in the tissue and transported to the upper compartment.

## Discussion

The bile duct enhances the performance of in vitro liver tissue as it specifically collects the bile [1]. Bile is an emulsifier, stores chemical and drug metabolites, and indicates the presence of certain diseases [3, 5, 35]. Here, we constructed an enhanced in vitro tubular bile duct structure in a triple co-culture system mimicking IHBD morphogenesis in rat embryos. This structure showed superior function to the standard bile duct cyst structure. Additionally, we established an open-culture system supporting the interaction between the bile duct and the hepatocyte tissue. This system exhibited autologous metabolite transport and accumulation from hepatocytes, enhancing the feasibility of in vitro bile recovery for high-throughput cell-based assays or use as relevant disease models.

Our triple-hierarchical co-culture model successfully activated three cell types instrumental to in vitro bile duct establishment by reproducing IHBD morphogenesis. Our results underscore the fact that hepatoblasts are developed and organized as a major part of the ductular structure, whereas BECs and MEFs act as supportive liver cells in establishing a tubular bile duct model and may support subsequent signaling processes. Cultures containing only hepatoblasts in ECM–embedded or ECM overlay tended to form more cysts than elongated structures. In addition, MEFs and BECs attached to the flat PDMS surface determined the elongated shape of the bile duct early in differentiation. The subsequently formed structures were enlarged and retained their initial shape rather than becoming elongated and branched. Although the detailed cells composition in the tubular structure remains unclear at present, our data strongly suggested that the cell-cell interaction between hepatoblasts and BECs/MEFs is required for the process of tubule formation whereas the hepatoblasts themselves are a major cell source for the duct structure.

The In vivo IHBDs emerged from the hepatic portal veins [17, 18] and flat PDMS surfaces maintained MEF and BEC growth in a manner similar to that observed in the hepatic portal vein. Thus, MEFs and BECs induced the bile duct to form an elongated structure. During tubular bile duct establishment, *Jagged1*-*Notch2* activation was relatively higher than in the cyst, indicating superior tubulogenesis in the tubular formation. In terms of bile duct organization [26], the tubular also maintained better bile duct organization than in the cyst as determined by higher *Cldn15* expression. However, the mechanism of *Cldn15* activation and its relationship with *Jagged1-Notch2* signaling have not been investigated. It has been hypothesized that the IHBD is generated from the conjunction of cysts [17, 18]. Our reconstructed morphogenesis suggests that these observed cysts arise as a consequence of the discrete rate of differentiation in the mound structure.

As the in vitro bile duct requires spatial support to form a 3D structure [30], the overlaid ECM technique can also support bile duct establishment and provide an open-culture system. The overlaid ECM technique frees one side of the ECM for hepatobiliary interaction. In contrast, the ECM–embedded environment occupies the entire culture system. Our data confirms that a high Matrigel content is conducive to in vitro bile duct differentiation. This is because Matrigel is mainly composed of laminin-1, which activate the β1 integrin signals essential for apicobasal polarity maintenance [9, 42], whereas the inclusion of collagen type I additionally supports bile duct-like structure branching [43]. Ideal mixtures combining these two ECMs are proven to synergistically enhance tubular structure formation. However, the use of high laminin concentrations in our study did not alter tubular bile duct formation as the ECM mixture could not sustain tissue attachment resulting in data incomparable to the ideal ECM mixture. Considering that reduced growth factor (GF)-Matrigel still contains GFs, including TGF–β1, the sparse concentration of possible TGF–β1 may have influenced the establishment process. The TGF–β1 concentration under all culture conditions was detected at up to 20 pg mL^− 1^ over 17 days (Additional file 6; Fig. S6). Based on this information, TGF–β1 is a robust cytokine that is only required at low concentrations for bile duct differentiation and has less influence on tubular bile duct formation.

The tubular bile duct structure established in the triple co-culture system functioned more effectively than the standard cyst structure. Transporter activity indicated that the functions related to bile transport and drug response were positively regulated. We also validated that specific genes have relatively less influence on structure and function but have greater involvement in regulating bile duct differentiation such as *Sox9* and *Ck19.* Immunofluorescence staining revealed that VIM was expressed in certain tubular bile duct structures indicating an epithelial-mesenchymal transition (EMT) morphology. EMT is characteristic of structures configured by flat-type BEC and is indicative of progressive hepatic fibrosis [28]. However, our EMT-expressing tubular structure displayed growth and function like those of non-EMT-expressing bile ducts. Thus, the presence of TGF−β1 is not only vital for BEC differentiation, but also triggers hepatic fibrosis [28, 36].

In addition to its tubular shape, our in vitro bile duct can perform separate autologous metabolite analog recovery from hepatocyte culture and thus can be utilized for bile metabolite collection in vitro. The absence of BEC affected fluorescein secretion from the hepatocyte bile canaliculi to the culture medium in the lower compartment, whereas the BEC monolayer facilitated fluorescein transportation to the culture medium in the upper compartment. In contrast to the BEC monoculture, fluorescein accumulated in the lumen of the tubular structure after being concentrated in the hepatocyte bile canaliculi. Unlike in the liver lobules, the in vitro bile duct–hepatocyte interaction did not indicate a direct connection between two tissues. We presume that the fluorescein in the bile canaliculi passively leaked and was readily recovered by the tubular bile duct structure through transporters. Considering that highly concentrated metabolites can be recovered separately from hepatocytes via bile duct tissue, our liver culture model is a notably functional and practical method with potential for in vitro primary bile recovery. The culture separation is ideal for long term in vitro hepatocytes and useful for clearly analyzing bile transportation and accumulation between two cellular components. Going forward, further assays to collect primary bile and drug metabolites are important for improving this model. This ability is also useful for long term cell-based hepatotoxicity assays by reducing the toxicity impact of accumulated bile [3]. Direct contact in hepatobiliary tissue corresponding to the physiological canals of Hering may improve transport and metabolite accumulation. Using this co-culture technique, observation of bile duct morphogenesis can help simplify parameters in developmental studies, and thus be more manageable and accurate than the animal model. The tubular bile duct structure may also be useful for forming a bile duct network for biliary disease treatment and transplantation.

## Conclusion

In vitro bile recovery may serve as an advanced cell-based chemical and drug assay. We developed an enhanced bile duct culture system for separate liver metabolite recovery from liver culture. This triple co-culture system is practical and effective in establishing a tubular bile duct structure for in vitro bile recovery. Moreover, its ability to separate metabolite recovery may support its integration with biodevices and broaden the applicability and practicality of hepatobiliary culture. We confirmed the reproducibility of in vitro bile duct morphogenesis and contributed to the understanding of developmental biology. An enhanced-tubular bile duct structure also may also improve the performance of the bile duct model in assessing biliary diseases and transporter activity.

## Material and methods

### Plate culture surface coating

The surface of a PDMS-bottom 96-well plate [24, 37] was used and treated with 3-mercaptopropyltrimethoxysilane-GMBS cross-link surface treatment [23]. The PDMS surface reacted with the 3-mercaptopropyltrimethoxysilane 4% w/v after oxygen plasma treatment and was the subjected to 0.28% w/v *N*-(4-maleimidobutyryloxy) succinimide (GMBS) cross-link. The surface was then activated with 1-ethyl-3-(3-dimethylaminopropyl) carbodiimide, hydro-chloride/WSC (Dojindo Molecular Technologies Inc., Rockville, MD, USA) and *N*-hydroxysulfosuccinimide sodium salt/Sulfo-NHS (TCI, Tokyo, Japan). Collagen type I coating (1%) (Nitta Gelatin NA, Morrisville, NC, USA) was applied after overnight incubation of the cross-link surface in phosphate-buffered saline (PBS).

### Primary hepatoblast isolation

Rat primary hepatoblasts cells were isolated from E.17.5 female Wistar rats (CLEA Japan, Inc., Tokyo, Japan) using previously published protocols [1, 38] with minor modifications. Briefly, fetal livers were minced in perfusion buffer at 37 °C for 10 min. They were then lysed in the liver digestion medium (Invitrogen, Carlsbad, CA, USA) at 37 °C for 20 min and centrifuged at 1200× *g* and 4 °C for 3 min. The cells were strained 3× through a 40–μm mesh Falcon® cell strainer (Corning Inc., Corning, NY, USA) followed by two or three washings with 3% v/v fetal bovine serum-phosphate-buffered saline (FBS-PBS) at 700× *g* and 4 °C for 3 min. Hepatoblasts with ≥ 85% viability were used for culture. FACS analyses indicated ≥ 75% purity for the hepatoblasts isolated (Additional file 7; Fig. S7). The hepatoblasts were maintained in hepatoblast medium [1] modified as follows: William’s E medium (Thermo Fisher Scientific, Waltham, MA, USA) supplemented with 10% FBS (Biosera, Nuaille, France), 1 mM MEM-nonessential amino acids (Thermo Fisher Scientific, Waltham, MA, USA), 10 mM nicotinamide (Sigma-Aldrich Corp., St. Louis, MO, USA), 0.2 mM ascorbic acid (Wako Pure Chemicals Industries, Ltd., Osaka, Japan), 50 μg mL^− 1^ gentamycin (Wako Pure Chemicals Industries, Ltd., Osaka, Japan), 1 × insulin inhibitor solution (Thermo Fisher Scientific, Waltham, MA, USA), 10^− 7^ dexamethasone, and 10 ng mL^− 1^ mouse epidermal growth factor (mEGF) (Sigma-Aldrich Corp., St. Louis, MO, USA).

### Primary intrahepatic BEC isolation

Primary intrahepatic BEC was isolated from 6-wk male Wistar rats (CLEA Japan, Inc., Tokyo, Japan). Primary intrahepatic BEC were obtained by a modified standard two-step collagenase liver perfusion protocol [23] followed by differential centrifugation of the remaining hepatocyte-free supernatant [1, 38] with modifications. Briefly, hepatocyte-free supernatant was centrifuged at 700× *g* and 4 °C for 2 min to remove any remaining hepatocytes. The hepatocyte-free supernatant was recentrifuged at 1800× *g* and 4 °C for 3 min. Dead cells and red blood corpuscles were discarded using Percoll and hemolysis buffer. The BECs were washed and centrifuged at 1200× *g* and 4 °C for 3 min. Cells were strained through a 40–μm mesh Falcon® cell strainer (Corning Inc., Corning, NY, USA) before subculturing. FACS analyses indicated ≥ 70% purity of the isolated IHBC isolated (Additional file 7; Fig. S7).

For the in vitro model, primary intrahepatic BEC passaging was required to improve the proliferation rate and purity. Differential trypsinization was applied for 3 min and again for 5 min to remove any hepatoblasts and stellate cells that could potentially grow in the subsequent passages. The resultant passaged was used in the following experiments and cultured in oval cell medium [38] containing William’s E medium (Thermo Fisher Scientific, Waltham, MA, USA) supplemented with 10% FBS (Biosera, Nuaille, France), 2 mM *L*-glutamine (Thermo Fisher Scientific, Waltham, MA, USA), 10 mM nicotinamide (Sigma-Aldrich Corp., St. Louis, MO, US), 0.2 mM ascorbic acid (Wako Pure Chemicals Industries, Ltd., Osaka, Japan), 20 mM HEPES (Sigma-Aldrich Corp., St. Louis, MO, US), 1 mM Na-pyruvate (Thermo Fisher Scientific, Waltham, MA, USA), 14 mM *L*-glucose (Sigma-Aldrich Corp., St. Louis, MO, US), 0.15% w/v sodium hydrogen carbonate (NaHCO_3_), and 50 μg mL^− 1^ of gentamycin (Wako Pure Chemicals Industries, Ltd., Osaka, Japan). The supplementation mixture for the aforementioned medium consisted of 1 × insulin inhibitor solution (Thermo Fisher Scientific, Waltham, MA, USA), 10^− 7^ dexamethasone, 1 × ROCK inhibitor/Y27632, 10 10 ng mL^− 1^ mouse hepatocyte growth factor (HGF), and 10 ng. mL^− 1^ mEGF (Sigma-Aldrich Corp., St. Louis, MO, US) and was added immediately before use.

### Primary hepatocytes isolation

Primary hepatocyte was isolated from 6-wk male Wistar rats (CLEA Japan, Inc., Tokyo, Japan). Hepatocytes were obtained by the same method as that used for primary intrahepatic BEC isolation [23]. However, the remaining hepatocyte pellets were washed thrice with culture medium followed by centrifugation at 600×* g* and 4 °C for 1 min with minimum acceleration and deceleration. A mixture up to 22.5 mL Percoll with 2.5 mL HBSS 10× (Thermo Fisher Scientific, Waltham, MA, USA) was added to remove dead cells. Hepatocytes with ≥ 80% viability and ≥ 98% purity (Additional file 7; Fig. S7) were used for culture.

### FACS analyses

Isolated primary cells were fixed with 4% w/v paraformaldehyde (PFA) for 20 min. After two PBS washings, cold methanol was added and the sample was incubated at − 20 °C for 7 min. The primary ab was added after a subsequent PBS wash, incubated overnight at 4 °C, and incubated with the secondary ab conjugated to Alexa Fluor-555 for 2 h at 20–25 °C. in the dark. Samples were measured with a MUSE® cell analyzer and InCyte™ MUSE® analyzer 3.3 software (EMD Millipore, Billerica, MA, USA).

### Bile duct-like structure establishment

A hierarchical co-culture was set up with MEF feeder cells (Cell Biolabs, Inc., San Diego, CA, USA), BECs, and hepatoblasts incubated on PDMS-coated bottom of a 96-well cross-linked collagen plate as previously described (Fig. 1a). To inhibits proliferation, MEF was treated with 10 μM final concentration of mitomycin C at 37 °C for 2 h. Treated MEF and intrahepatic BEC were mixed in equal proportions and seeded at a density of 2 × 10^5^ cm^− 2^. The hepatoblasts were then added at a seeding density of 1.5 × 10^6^ cm^− 2^. The ECM overlay was conducted 1 d after hepatoblast seeding. PBS washing was performed to remove dead hepatoblasts prior to ECM overlay. The following ECM mixtures were prepared: Matrigel-reduced growth factor (final concentration 9.5 mg mL^− 1^, Corning Inc., Corning, NY, USA) - collagen type I gel (Nitta Gelatin NA, Morrisville, NC, USA), 9:1; Matrigel-collagen type I gel, 1:1; and laminin high concentration (3.5 mg mL^− 1^, Corning Inc., Corning, NY, USA) - collagen type I gel, 3:2 [9]. All cultures containing hepatoblasts were raised on hepatoblast culture medium which was replaced every other day.

### Surface area measurement

Images were collected two samples and three independent experiment as described. The bile duct-like structure surface area was calculated in ImageJ 1.48u4 software (NIH, Bethesda, MD, USA) (Additional file 1; Fig. 1b) and the converted to a percentage.

### Integrated bile duct-hepatocyte culture model

The tubular bile duct structure established from the previous model was co-cultured with primary rat hepatocyte using a 24-well plate AteloCell® permeable membrane-culture insert (Koken Co. Ltd., Tokyo, Japan). Before seeding, PDMS discs were inserted to prevent collagen membrane creasing (Additional file 6; Fig. 6a). After day 11 of tubular establishment, hepatocytes were seeded on the opposite side of the collagen membrane at a density 2 × 10^5^ cm^− 2^. The culture insert was transferred to the PDMS-coated bottom of a 24-well plate with hepatocytes placed on the lower compartment and incubated with hepatocyte medium containing William’s E medium supplemented with 0.1 μM ITS (Thermo Fisher Scientific, Waltham, MA, USA), 10 μM mEGF (Sigma-Aldrich Corp., St. Louis, MO, US), 20 mM HEPES, 0.5 mM ascorbic acid, and 1× penicillin-streptomycin-Amphotericin (Wako Pure Chemical Industries, Ltd., Osaka, Japan). The medium was enriched with 300 μg mL^− 1^ Matrigel [23]. The culture medium was changed and collected every other day for 10 d and used in the subsequent analyses.

### Immunostaining and histomorphological remodeling

Immunostaining analysis was conducted according to a previously reported protocol [6] with minor modifications. Briefly, the ECM was removed using 1000 U mL^− 1^ collagenase (Wako Pure Chemical Industries, Ltd., Osaka, Japan) and incubated for 10 min as previously described [39]. The samples were fixed with 4% PFA for 20 min, permeabilized with 0.1% v/v Triton X for 15 min, and blocked with gelatin buffer for 1 h. The primary ab was added and incubated overnight at 4 °C. The secondary ab was added and incubated for 3 h (Additional file 8; Table S8). For histomorphological remodeling during bile duct-like structure development, phalloidin-conjugated Alexa 647 (Santa Cruz Biotechnology, Dallas, TX, USA) staining was performed to reconstruct the actin filament. Samples were stained with phalloidin 1 × dissolved in PBS with 1% w/v bovine serum albumin (BSA) for 3 h followed by fixation with 4% PFA. Hoechst (Dojindo Molecular Technologies Inc., Rockville, MD, USA) nuclear stain 1/200 × in PBS, was added for 5 min at the end of PBS washing. Samples were observed under a Fluoview FV3000 confocal microscope (Olympus, Tokyo, Japan) with normalized laser and color strength. The 3D-stacked image series was analyzed by FV3000 image processing (Olympus, Tokyo, Japan) and iQuant image analyzer [40] software.

### Bile analog transportation

The activity of multidrug resistance protein 1 (MDR1) was assessed by rhodamine 123 efflux with or without verapamil [7, 9, 30]. A 20–μM verapamil (BD Bioscience, Kanagawa, Japan) treatment was administered at 37 °C for 20 min prior to rhodamine 123 addition. The bile duct-like structures were washed and incubated with 100 μM rhodamine 123 (BD Bioscience, Kanagawa, Japan) in serum-free culture medium at 37 °C for 15 min. The cultures were then rinsed twice with fresh medium. Fluorescence dye accumulation was recorded by confocal microscope (Olympus, Tokyo, Japan) under a rhodamine green (502–527 nm) emission spectrum. For the inserts, 5 μg mL^− 1^ fluorescein diacetate (FDA) (Sigma-Aldrich Corp., St. Louis, MO, USA) [1, 33] was added to the bottom compartment of the hepatocyte culture and incubated at 37 °C for 70 min. The FDA was replaced with fresh culture medium which was then incubated at 37 °C for 40 min. FDA transport was detected under a fluorescein isothiocyanate (FITC) (495–519 nm) emission spectrum. FITC was recorded over 110 min at 10-min intervals. Samples were drawn from the culture medium and measured with a NanoDrop 3300 spectrophotometer (Thermo Fisher Scientific, Waltham, MA, USA). A pure FITC/FDA solution was used as the calibration standard. To measure the fluorescein content, the tissue was dissolved in DMSO and compared to a DMSO solution of FITC, which used to obtain the standard curve. The fluorescein concentrations were interpolated from the exponential curve of the reference standard absorbance.

### Qualitative gene expression assays

Tissue RNA and cDNA samples were obtained using previously reported methods [30]. Briefly, after collecting samples with RNA concentrations100 μg mL^-l^, reverse transcription (RT)-PCR was run to capture the cDNA with PrimeScript™ RT Master Mix-Perfect Real Time (TaKaRa Bio, Inc., Shiga, Japan) protocols and the following phases: 37 °C, 15 min; 50 °C, 5 min; 98 °C, 5 min; and 4 °C, hold. The expression of biliary cell-related function was evaluated by quantitative real-time (qRT)-PCR using several specific biliary markers followed by cDNA amplification with KOD SYBR qPCR mix (Toyobo Co., Ltd., Osaka, Japan) in a qRT-PCR system (StepOnePlus; Thermo Fisher Scientific, Waltham, MA, USA). Fifteen markers (Eurofins Scientific, Brussels, Belgium) (Additional file 9: Table S9) were used to assess gene expression in the bile duct-like structure. *β*-actin served as a housekeeping gene. The following cycling conditions were used: pre-denaturation at 98 °C for 60 s; five cycles of denaturation at 98 °C for 15 s; annealing-extension at 68 °C for 30 s; 40 cycles of denaturation at 98 °C for 15 s; annealing at 60 °C for 10 s; and a final extension at 68 °C for 30 s.

### Statistical analyses

ANOVA was conducted for each independent experiment and applied to the bile duct-like structure surface area. Data were means ± SD. For qRT-PCR, relative gene expressions were calculated by comparative ∆∆ Ct for several independent experiments. All data were analyzed by a two-way Student’s *t*-test to identify significant differences between sample means. Statistical analyses were performed with Microsoft Excel.

## Supplementary information

**Additional file 1: Fig. S1.** Optimum culture condition for bile duct structure establishment mimicking IHBD morphogenesis. **a,** In vitro bile duct-like development on polystyrene (TCPS) and PDMS plate. Image taken from triple co-culture − overlay optimum ECM at day 11. **b,** Morphology observed in culture variation. Bile duct-like structures dominated in hepatoblast containing culture. This image was used as to determine bile duct structure surface area (mm^2^) for bile duct map making. **c,** Complete bile duct structure surface area (mm^2^) map including culture medium modulations #1, #2 [18], and #3 [41]. **d,** Sinusoidal structure observed in the triple co-culture−overlay Matrigel: collagen, 1:1 culture. Immunofluorescence-stacked images show ALB and VIM expression and emphasize lack of 3D formation.

**Additional file 2: Fig. S2.** Rhodamine 123 transport recorded within 15 min in the absence or presence of verapamil**.** Images were taken every 2 min (except at t = 10–15 min) from triple co-culture−overlay optimum ECM at day 11.

**Additional file 5: Fig. S5.** Integrated bile duct-hepatocyte culture using a collagen membrane culture insert. **a,** Seeding process on the upper and lower compartments of the culture insert. A PDMS disk was applied to increase the surface tension on the collagen membrane. In lower compartment seeding, the culture medium behaved in a manner similar to the PDMS disk and maintained the viability of the cultured tissue which developed on the upper compartment during the preceding days. **b,** Fluorescence change in FDA in the culture insert was observed for 110 min incubation at 10-min intervals. Total fluorescence between the upper and lower compartments remained stable after 20–30 min incubation. **c,** Fluorescein transport on the lower and upper compartments. All treatment variables are compared. Integrated culture combining tubular bile duct and hepatocyte culture dominate fluorescein retention. Yellow backlights represent FDA removal from the lower compartment (*n* = 4; two independent experiments).

**Additional file 6: Fig. S6.** TGF*-*β1 concentration trend including culture medium modulations #1, #2, and #3. High TGF*-*β1 concentrations in BECs differentiation medium inhibit hepatoblast differentiation into BECs.

**Additional file 7: Fig. S7.** FACS analyses of primary rat hepatocyte, hepatoblast, and intrahepatic BECs**.** The markers used were ALB for hepatocytes, AFP for hepatoblasts, and EpCAM for epithelial cells. Primary hepatocytes showed ~ 98% purity. Primary hepatoblasts had ~ 75% purity with ~ 10% epithelial cell contamination. Intrahepatic BECs were ~ 75% pure after two passages.

**Additional file 8: Table S8.** Antibodies and chromophores used in immunostaining.

**Additional file 9: Table S9.** Primers used in qRT-PCR.

## Data Availability

All data generated or analyzed during this study are included in this published article [and its supplementary information files]. More details data are available from the corresponding author on reasonable request.

## Abbreviations

BEC: Biliary epithelial cell
BSA: Bovine serum albumin
DMSO: Dimethyl sulfoxide
ECM: Extracellular matrix
ELISA: Enzyme-linked immunosorbent assay
EMT: Epithelial-mesenchymal transition
FBS: Fetal bovine serum
FDA: Fluorescein diacetate
FITC: Fluorescein isothiocyanate
GF: Growth factor
HBSS: Hanks balanced salt solution
HGF: Hepatocyte growth factor
IHBD: Intrahepatic biliary duct
iPS: Induced pluripotent stem cells
MDR: Multidrug resistance protein
MEF: Mouse embryonic fibroblast
PBS: Phosphate-buffered saline
PDMS: Polydimethylsiloxane
PFA: Paraformaldehyde
qRT-PCR: Quantitative real-time polymerase chain reaction
RT-PCR: Reverse-transcription polymerase chain reaction
TCPS: Tissue culture polystyrene
TGF: Transforming growth factor
