# Supplementary figures and images for: Tubular bile duct structure mimicking bile duct morphogenesis for prospective in vitro liver metabolite recovery
Source: J Biol Eng. 2020 Mar 19;14:11. doi: 10.1186/s13036-020-0230-z (PMC7081557; doi:10.1186/s13036-020-0230-z)

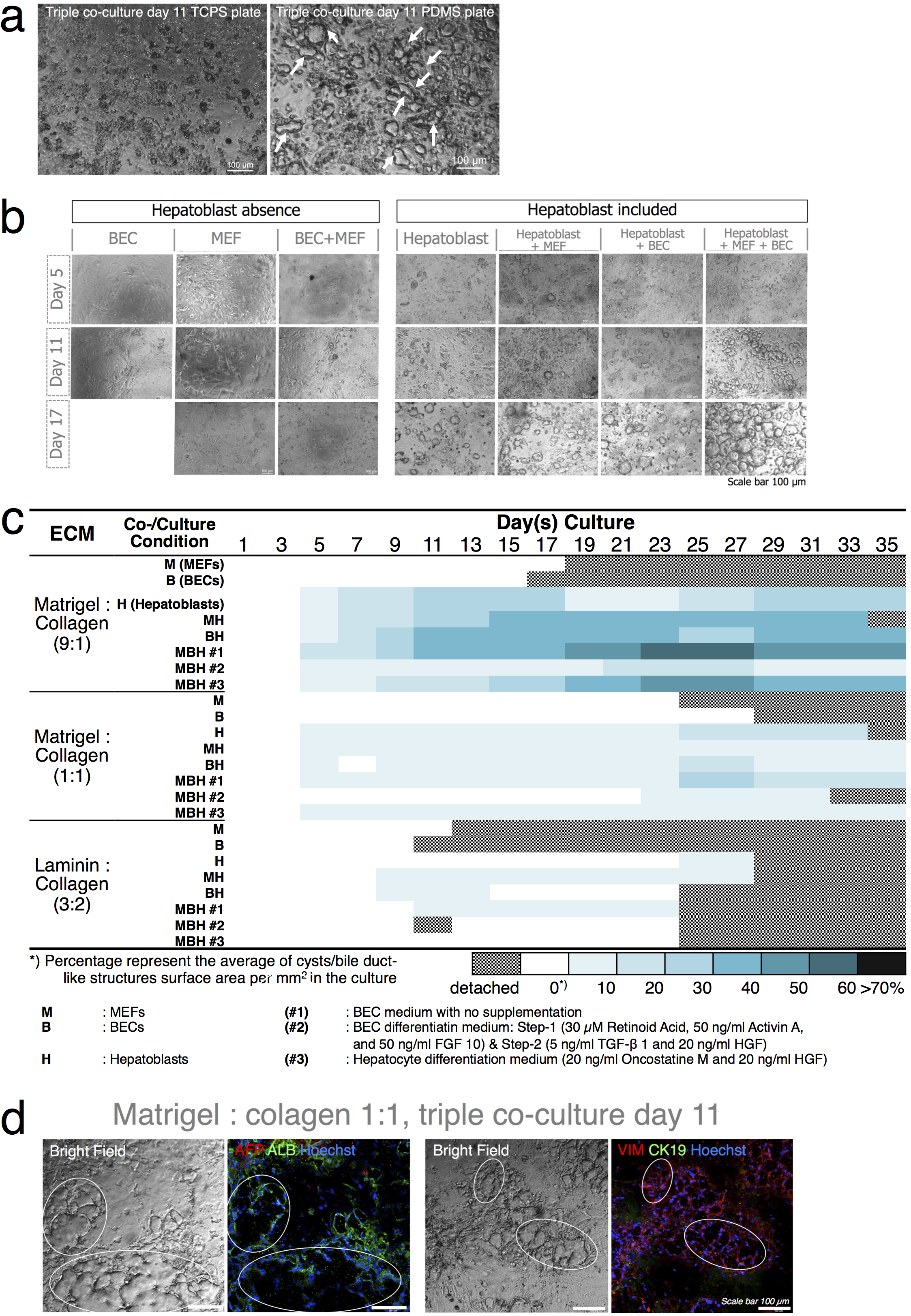

Supplement: Supplementary file 3 — Additional file 1: Fig. S1. Optimum culture condition for bile duct structure establishment mimicking IHBD morphogenesis. a, In vitro bile duct-like development on polystyrene (TCPS) and PDMS plate. Image taken from triple co-culture − overlay optimum ECM at day 11. b, Morphology observed in culture variation. Bile duct-like structures dominated in hepatoblast containing culture. This image was used as to determine bile duct structure surface area (mm2) for bile duct map making. c, Complete bile duct structure surface area (mm2) map including culture medium modulations #1, #2 [18], and #3 [41]. d, Sinusoidal structure observed in the triple co-culture−overlay Matrigel: collagen, 1:1 culture. Immunofluorescence-stacked images show ALB and VIM expression and emphasize lack of 3D formation. [file 13036_2020_230_MOESM1_ESM.png]

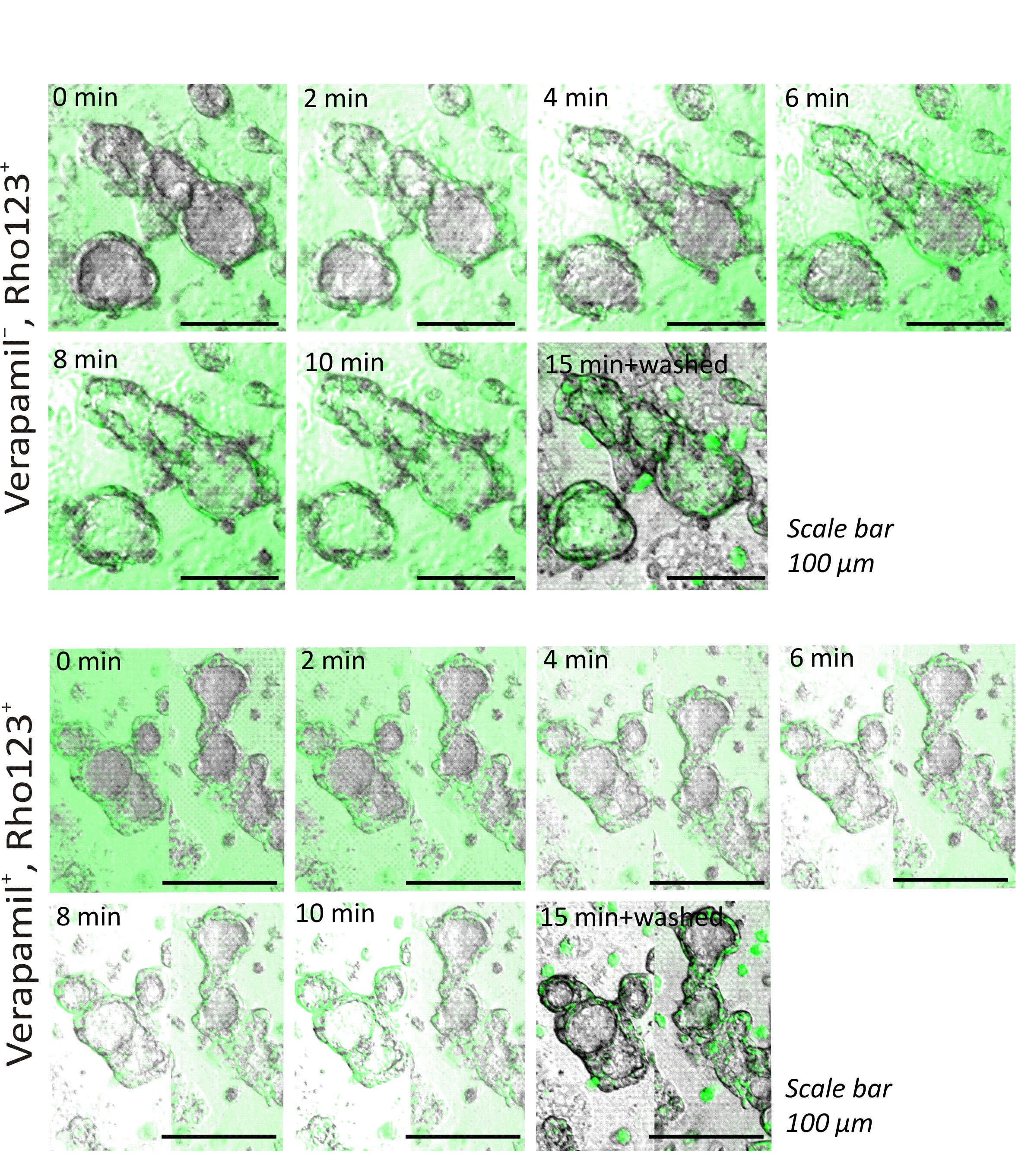

Supplement: Supplementary file 4 — Additional file 2: Fig. S2. Rhodamine 123 transport recorded within 15 min in the absence or presence of verapamil. Images were taken every 2 min (except at t = 10–15 min) from triple co-culture−overlay optimum ECM at day 11. [file 13036_2020_230_MOESM2_ESM.png]

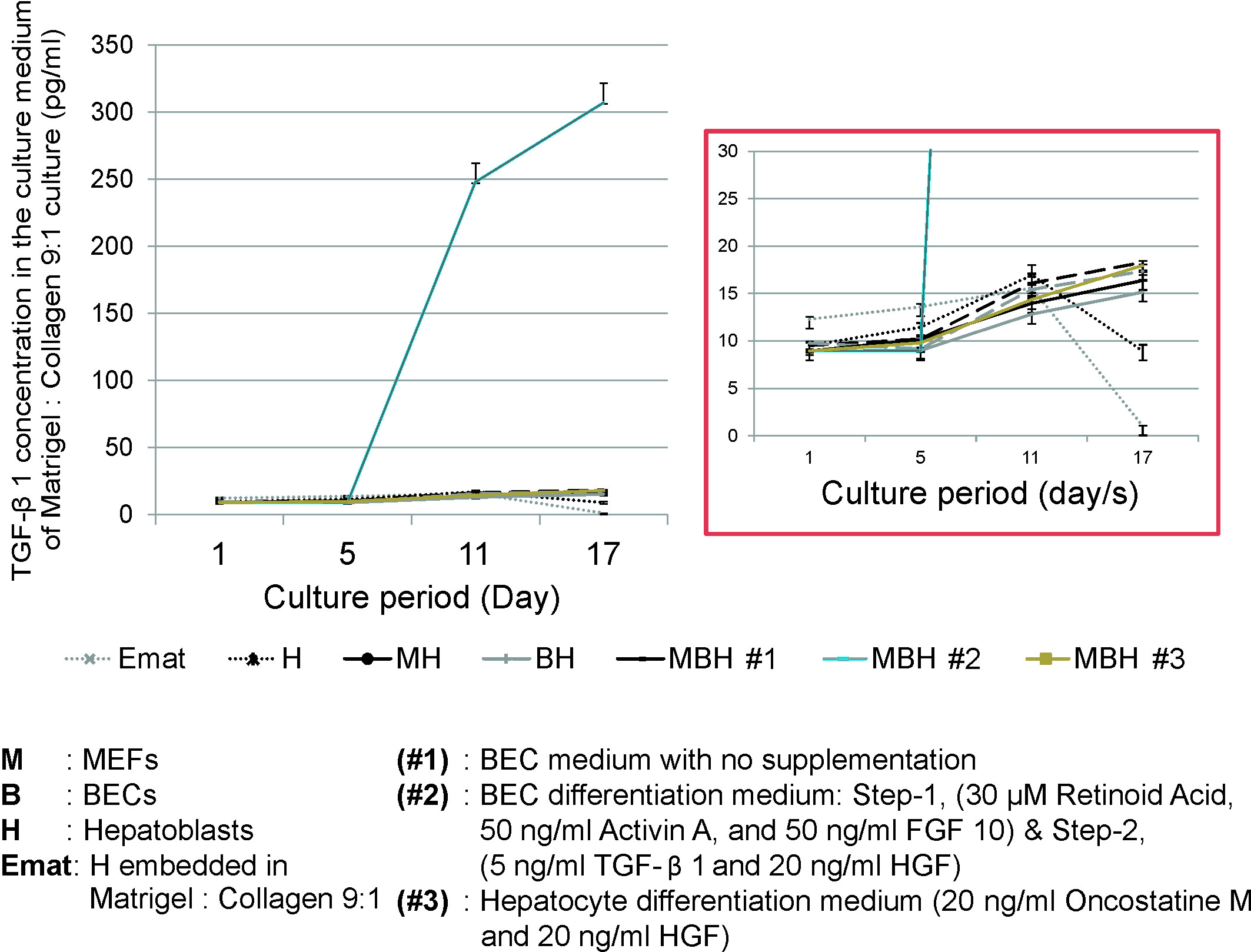

Supplement: Supplementary file 5 — Additional file 5: Fig. S5. Integrated bile duct-hepatocyte culture using a collagen membrane culture insert. a, Seeding process on the upper and lower compartments of the culture insert. A PDMS disk was applied to increase the surface tension on the collagen membrane. In lower compartment seeding, the culture medium behaved in a manner similar to the PDMS disk and maintained the viability of the cultured tissue which developed on the upper compartment during the preceding days. b, Fluorescence change in FDA in the culture insert was observed for 110 min incubation at 10-min intervals. Total fluorescence between the upper and lower compartments remained stable after 20–30 min incubation. c, Fluorescein transport on the lower and upper compartments. All treatment variables are compared. Integrated culture combining tubular bile duct and hepatocyte culture dominate fluorescein retention. Yellow backlights represent FDA removal from the lower compartment (n = 4; two independent experiments). [file 13036_2020_230_MOESM5_ESM.jpg]

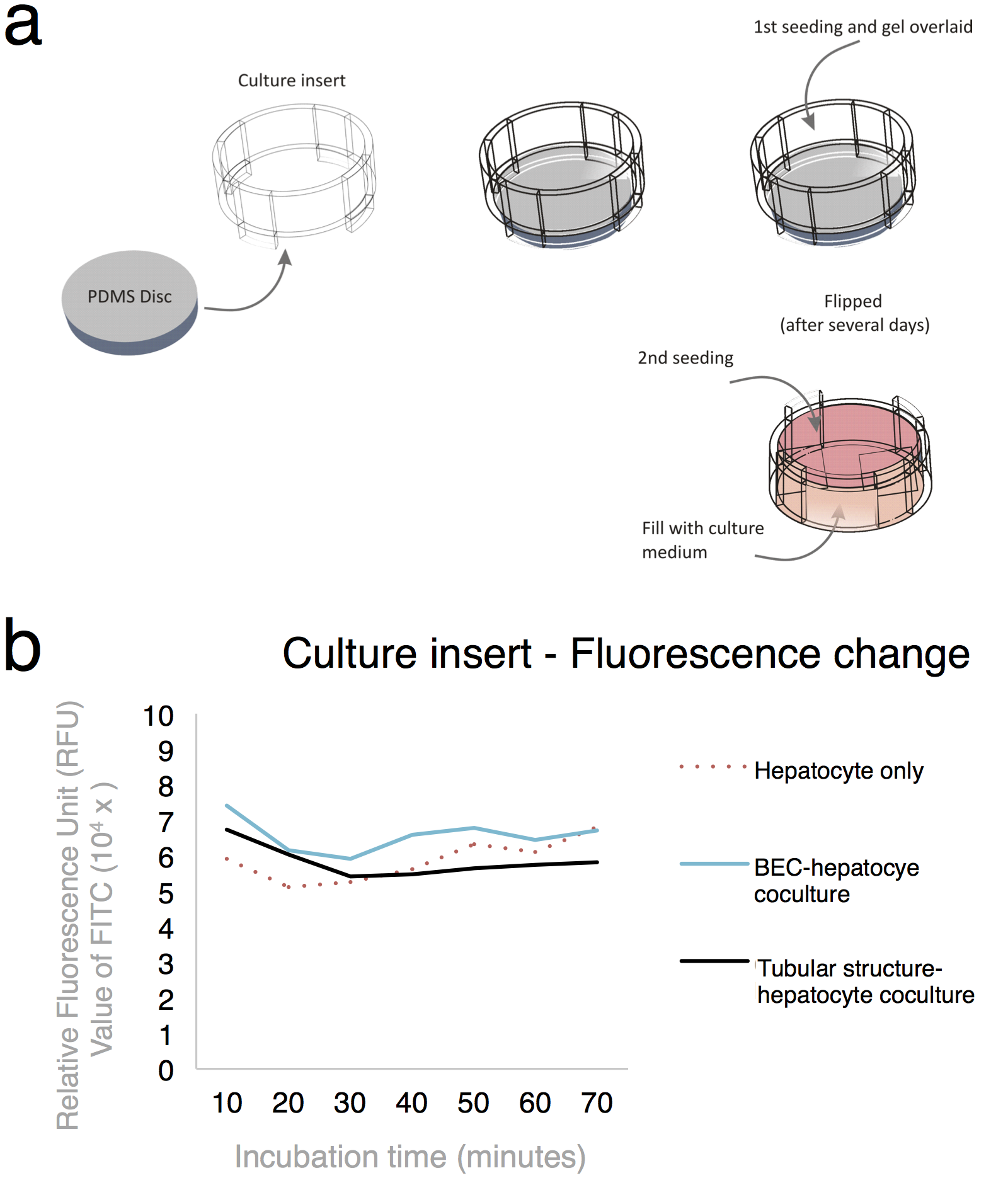

Supplement: Supplementary file 6 — Additional file 6: Fig. S6. TGF-β1 concentration trend including culture medium modulations #1, #2, and #3. High TGF-β1 concentrations in BECs differentiation medium inhibit hepatoblast differentiation into BECs. [file 13036_2020_230_MOESM6_ESM.png]

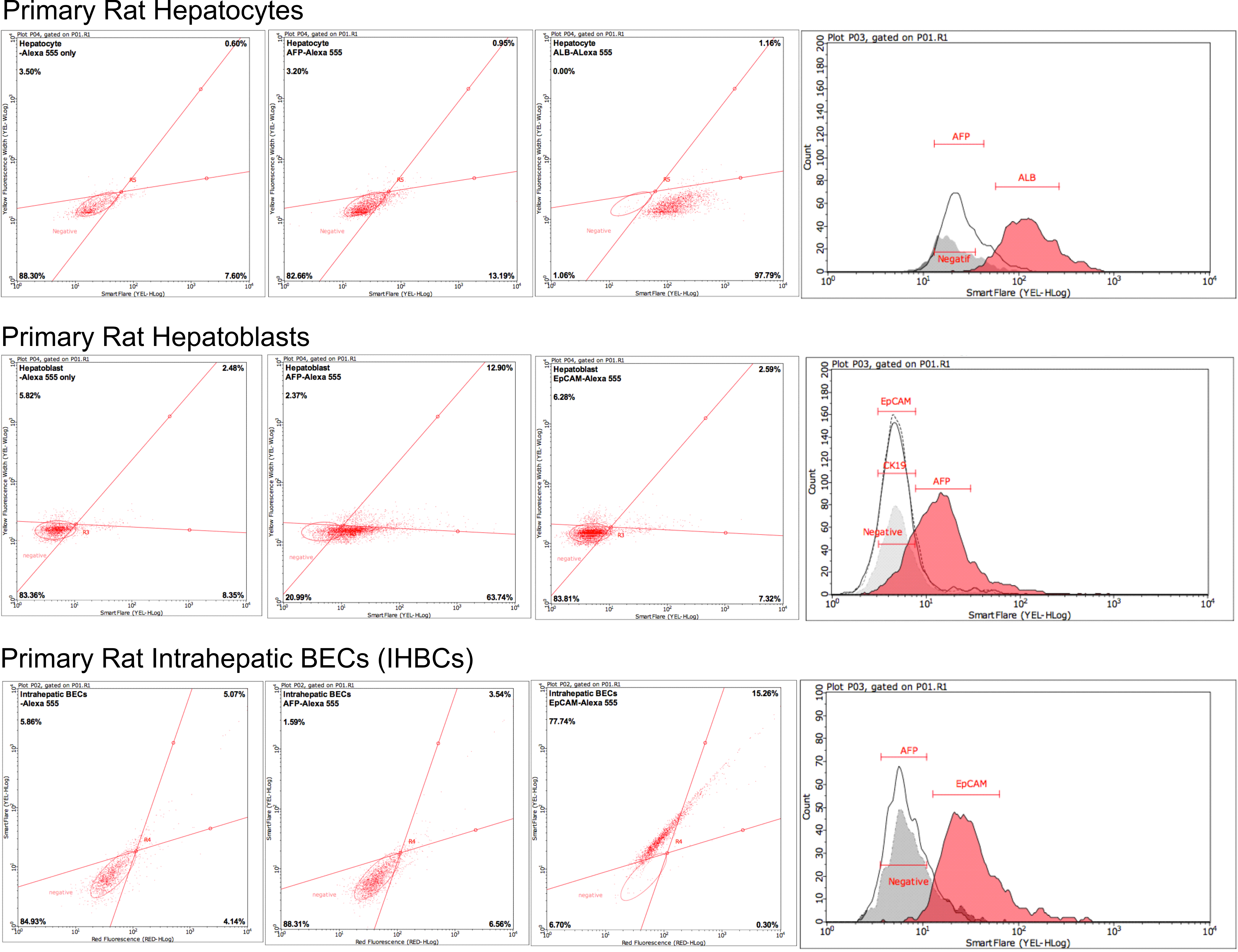

Supplement: Supplementary file 7 — Additional file 7: Fig. S7. FACS analyses of primary rat hepatocyte, hepatoblast, and intrahepatic BECs. The markers used were ALB for hepatocytes, AFP for hepatoblasts, and EpCAM for epithelial cells. Primary hepatocytes showed ~ 98% purity. Primary hepatoblasts had ~ 75% purity with ~ 10% epithelial cell contamination. Intrahepatic BECs were ~ 75% pure after two passages. [file 13036_2020_230_MOESM7_ESM.png]
