# Supplementary material for: Tubular bile duct structure mimicking bile duct morphogenesis for prospective in vitro liver metabolite recovery
Source: J Biol Eng. 2020 Mar 19;14:11. doi: 10.1186/s13036-020-0230-z (PMC7081557; doi:10.1186/s13036-020-0230-z)
Supplement: Supplementary file 8 — Additional file 8: Table S8. Antibodies and chromophores used in immunostaining. [file 13036_2020_230_MOESM8_ESM.docx]

**Table S8. Antibodies and chromophores used in immunostaining.**

| **Name of antibodies** | | **Manufactures** | **Expressed by (type of cells)** |
| --- | --- | --- | --- |
| *1^st^ antibodies* | |  |  |
|  | α – rat AFP goat – poly | Santa Cruz | Hepatoblast  Immature BEC |
|  | α – rat CK19 mouse – mono | Elabscience | BEC (immature and mature) |
|  | α – rat SOX9 rabbit – mono | Abcam | Immature BEC |
|  | α – rat CFTR rabbit – mono | Abcam | Mature BEC |
|  | α – rat AE2 rabbit – poly | LSBio | Mature BEC |
|  | α – rat ALB rabbit – poly | GeneTex | Hepatocyte  Hepatoblast |
|  | α – rat ALB goat – poly | Santa Cruz |  |
|  | α – rat VIM rabbit – poly | Proteintech | Mesenchymal cells |
|  | α – rat ZO-1 rabbit – poly | ThermoFischer | Tight junction |
|  |  |  |  |
| *2^nd^ antibodies* | |  |  |
|  | α – goat Alexa 647 donkey | Invitrogen |  |
|  | α – rabbit Alexa 647 donkey |  |  |
|  | α – mouse Alexa 647 donkey |  |  |
|  | α – mouse Alexa 488 donkey |  |  |
|  | α – rabbit Alexa 488 donkey |  |  |
|  |  |  |  |
| *Conjugated fluorescence dye* | |  |  |
|  | Phalloidin – Alexa 647 | Santa Cruz | Actin filament |
|  |  |  |  |
| *Nucleus Staining* | |  |  |
|  | Hoechst | Dojindo | Nucleus |
