# Supplementary material for: Tubular bile duct structure mimicking bile duct morphogenesis for prospective in vitro liver metabolite recovery
Source: J Biol Eng. 2020 Mar 19;14:11. doi: 10.1186/s13036-020-0230-z (PMC7081557; doi:10.1186/s13036-020-0230-z)
Supplement: Supplementary file 9 — Additional file 9: Table S9. Primers used in qRT-PCR. [file 13036_2020_230_MOESM9_ESM.docx]

**Table S9. Primers used in qRT-PCR.**

| **No.** | **Primer** | **Gene Target** | **Sequences (Forward, Reverse)** | **Size (bp)** | **Accession Number** |
| --- | --- | --- | --- | --- | --- |
| 1 | *β-actin* | β-actin, conserved gene | 5’-GTCGTACCACTGGCATTGTG*-*3’  5’*-*TCTCAGCTGTGGTGGTGAAG*-*3’ | 180 | V01217.1 |
| 2 | *Alb* | Albumin | 5’*-*CCTTCCAGGAGAACCCTACC*-*3’  5’*-*GCTTTCTCTTTCACGGCATC*-*3’ | 188 | NM_134326.2 |
| 3 | *Afp* | Alpha-fetoprotein | 5’*-*ATGGAGTGCCTACAGGATGG*-*3’  5’*-*TTAAACCTTCCGGTTTGTCG*-*3’ | 196 | NM_012493.2 |
| 4 | *Ck19* | Cytokeratin/Keratin 19 | 5’-AGTAACGTGCGTGCTGACAC-3’  5’-AGTCGCACTGGTAGCAAGGT-3’ | 193 | NM_199498.2 |
| 5 | *Sox9* | SRY-Box 9 | 5’-CGTGTGGATGTCAAAGCAAC-3’  5’-TCTTGATGTGCGTTCTCTGG-3’ | 192 | NM_080403.1 |
| 6 | *Sctr* | Secretin receptor | 5’-CAGGGTCTGGTGGTAGCTGT-3’  5’-AGTGCTAGCCTTGGTGCTGT-3’ | 174 | NM_031115.1 |
| 7 | *Cftr* | cAMP-regulated chloride channel | 5’-CACCACTTGGAGCTGTCAGA-3’  5’-CCTAGCAAGACAGGCTGGAC-3’ | 221 | NM_031506.1 |
| 8 | *Ae2* | Anion exchange protein 2 | 5’-GAGCCCTTCTGCTGAAACAC-3’  5’-AGGAACACCACCGATGAGAG-3’ | 161 | NM_017048.2 |
| 9 | *Mrp3* | Multidrug resistance receptor 3 | 5’-GCTTATCCAGGCTCAAGACG-3’  5’-TCACACACAGGAGCCAGAAG-3’ | 234 | AF_072816.1 |
| 10 | *Aqp1* | Aquaporin 1 | 5’-CCGAGACTTAGGTGGCTCAG-3’  5’-TCATGCGGTCTGTAAAGTCG-3’ | 245 | NM_012778.1 |
| 11 | *Vim* | Vimentin | 5’-ACGAATACCGGAGACAGGTG-3’  5’-AGCAGGTCCTGGTATTCACG-3’ | 211 | NM_031140.1 |
| 12 | *Notch2* | Neurogenic locus notch homolog protein 2 | 5’-GCGAGTGTCTGAAGGGCTAC-3’  5’-CTGCCCATTGTTTACACACG-3’ | 200 | M93661.2 |
| 13 | *Jagged1* | Jagged 1 | 5’-ACCACTGAGCACATTTGCAG-3’  5’-TCCTTGACAGGGTTTCCATC-3’ | 167 | NM_019147.1 |
| 14 | *Cldn15* | Claudin 15 | 5’-ATACTTGCTGGAGCCTGTGG-3’  5’-CCAGGATGGAGAGCAGAGAG-3’ | 151 | NM_001107135.2 |
